# The *Arabidopsis thaliana* K^+^-Uptake Permease 5 (AtKUP5) Contains a Functional Cytosolic Adenylate Cyclase Essential for K^+^ Transport

**DOI:** 10.3389/fpls.2018.01645

**Published:** 2018-11-13

**Authors:** Inas Al-Younis, Aloysius Wong, Fouad Lemtiri-Chlieh, Sandra Schmöckel, Mark Tester, Chris Gehring, Lara Donaldson

**Affiliations:** ^1^Biological and Environmental Sciences and Engineering Division, King Abdullah University of Science and Technology, Thuwal, Saudi Arabia; ^2^College of Science and Technology, Wenzhou-Kean University, Wenzhou, China; ^3^Department of Neuroscience, University of Connecticut School of Medicine, Farmington, CT, United States; ^4^Department of Molecular and Cell Biology, University of Cape Town, Cape Town, South Africa

**Keywords:** AtKUP5, K^+^ transport, adenylate cyclase, cAMP, second messenger, *Arabidopsis thaliana*, cyaA, *trk1 trk2*

## Abstract

Potassium (K^+^) is the most abundant cation in plants, and its uptake and transport are key to growth, development and responses to the environment. Here, we report that *Arabidopsis thaliana* K^+^ uptake permease 5 (AtKUP5) contains an adenylate cyclase (AC) catalytic center embedded in its N-terminal cytosolic domain. The purified recombinant AC domain generates cAMP *in vitro*; and when expressed in *Escherichia coli*, increases cAMP levels *in vivo*. Both the AC domain and full length AtKUP5 rescue an AC-deficient *E. coli* mutant, *cyaA*, and together these data provide evidence that AtKUP5 functions as an AC. Furthermore, full length AtKUP5 complements the *Saccharomyces cerevisiae* K^+^ transport impaired mutant, *trk1 trk2*, demonstrating its function as a K^+^ transporter. Surprisingly, a point mutation in the AC center that impairs AC activity, also abolishes complementation of *trk1 trk2*, suggesting that a functional catalytic AC domain is essential for K^+^ uptake. AtKUP5-mediated K^+^ uptake is not affected by cAMP, the catalytic product of the AC, but, interestingly, causes cytosolic cAMP accumulation. These findings are consistent with a role for AtKUP5 as K^+^ flux sensor, where the flux-dependent cAMP increases modulate downstream components essential for K^+^ homeostasis, such as cyclic nucleotide gated channels.

## Introduction

As an essential macronutrient, K^+^ constitutes 6–10% of the plants’ dry weight (Evans and Sorger, 1966; Leigh and Jones, 1984) and plants maintain cytosolic K^+^ concentrations in the order of 100 mM (Macrobbie, 1987). As an important osmolyte, K^+^ is implicated in turgor-dependent volume regulation, for example stomatal pore movement (Kim et al., 2010; Hedrich, 2012; Jezek and Blatt, 2017) and cell expansion (Rigas et al., 2001; Elumalai et al., 2002). Amongst its many functions, K^+^ serves to compensate for negative electrical charges associated with proteins, organic acid anions and inorganic anions (Shabala and Pottosin, 2010) and it helps in the stabilization of cytosolic pH at around 7.2, a level optimal for most enzymatic reactions (Cuin and Shabala, 2007). Thus, cytosolic K^+^ homeostasis is crucial to optimal cell metabolism and the activity of various cytosolic enzymes involved in photosynthesis, oxidative metabolism, and protein synthesis (Marschner, 1995).

Plant cells have evolved sophisticated and efficient transport systems embedded in their plasma membranes which enable them to take up K^+^ (in its ionic form) from soils that are either rich (>1 mM) or poor in K^+^, where the concentrations can be in the μM range (Ashley et al., 2006; Grabov, 2007). Furthermore, K^+^ has to cross many tissues and barriers within the plant’s root system (epidermal and root hairs, stelar, and cortical cells) before reaching the xylem, from which it will be delivered to various tissues in the shoot (Ahmad and Maathuis, 2014; Wigoda et al., 2014).

Porters constitute the largest group of the Class 2 transporters which include uniporters, symporters, and antiporters (Busch and Saier, 2002). Uniporters, as their name implies, are unidirectional carrier proteins that transport ions passively down their difference in electrochemical potential. By contrast, both antiporter- and symporter-mediated processes must be energized, either by proton or sodium electrochemical potential differences, and therefore are capable of transporting ions even against a difference in electrochemical potential, as is often the case for K^+^. For this reason, symporters have been suggested to facilitate K^+^-acquisition from K^+^-poor soils (Maathuis et al., 1997; Rodrìguez-Navarro, 2000; Gierth and Maser, 2007).

The family of plant HAK/KUP/KT transporters have been described as electrochemical potential-driven transporters and are likely K^+^–H^+^ symporters (Rodrìguez-Navarro, 2000; Grabov, 2007; Scherzer et al., 2015). The plant K^+^ Transporters (KTs) were first identified through their homology to bacterial K^+^-Uptake Permeases (KUPs) and fungal High Affinity K^+^-Transporters (HAKs) (Schleyer and Bakker, 1993; Banuelos et al., 1995), hence their name. In plants, HAK/KUP/KT transporter genes have been found in evolutionarily diverse organisms ranging from green algae to angiosperms (Grabov, 2007). All but two of these proteins contain the typical 12 transmembrane spanning domains (Li et al., 2014; Very et al., 2014). A common feature shared by many of these transporters is their ability to complement K^+^-uptake in yeast or bacteria mutants that are defective in K^+^-uptake and thus they are regarded as K^+^-transporters (Very et al., 2014; Nieves-Cordones et al., 2016).

Plant HAK/KUP/KTs have previously been associated with K^+^-transport across membranes in *Arabidopsis thaliana* (Quintero and Blatt, 1997; Fu and Luan, 1998; Kim et al., 1998; Ahn et al., 2004; Han et al., 2016) and in important crops like barley (Santa-Maria et al., 1997) and rice (Banuelos et al., 2002; Horie et al., 2011; Li et al., 2014). Several of the *A. thaliana* HAK/KUP/KTs have been functionally characterized in *Escherichia coli* (triple mutants) and yeast (double mutants) lacking their native K^+^-uptake transporters and were found to complement the potassium transport deficiency. They have also been shown to be expressed in different tissues *in planta* when grown under sufficient K^+^ (1–2 mM) including the roots, leaves, siliques, and flowers (Kim et al., 1998; Ahn et al., 2004; Han et al., 2016). Ten of the thirteen genes that constitute this family are expressed in root hairs including *AtKUP5* (also referred to as *AtKT/KUP5*; AT4G33530), the subject of this investigation, and five are expressed in root tips. This highlights the significance of these transporters in K^+^-uptake. In fact, *AtKUP5* is exclusively and specifically expressed in root hairs (Ahn et al., 2004). Several genes of this family (*AtHAK5, 6*, *7*, and *8* and *AtKUP1, 2* and *3*) were found to be transcriptionally up-regulated upon K^+^-starvation (Kim et al., 1998; Rubio et al., 2000; Ahn et al., 2004). While some studies have shown that HAK/KUP/KT gene products are localized to the tonoplast (Jaquinod et al., 2007) and may be involved in vacuolar transport (Senn et al., 2001), at least one of these genes, *AtKUP7*, was shown to encode a plasma membrane protein involved in K^+^ uptake and translocation in roots under K^+^-limited conditions. Moreover, the loss of function of AtKUP7 leads to a reduction in K^+^-uptake rate and K^+^ content in xylem sap under K^+^-deficient conditions (Han et al., 2016).

It is particularly interesting that AtKUP7, a K^+^-transporter, was identified recently as a dual function (or moonlighting) protein. Indeed, AtKUP7 was found to harbor a functional adenylate cyclase (AC) catalytic domain that catalyzes the formation of cyclic adenosine 3′,5′-monophosphate (cAMP) from adenosine 5′-triphosphate (ATP) (Al-Younis et al., 2015). Cyclic AMP participates in key signal transduction pathways in all living organisms ranging from the simple prokaryotes to complex multicellular organisms (Yan et al., 2016; Gehring and Turek, 2017). In higher plants, cAMP has a role in many biological processes such as the activation of protein kinases in rice leaves (Komatsu and Hirano, 1993) and the promotion of cell division in tobacco BY-2 cells (Ehsan et al., 1998). More recently, cAMP has been implicated in plant stress responses and defense (Gottig et al., 2009; Lemtiri-Chlieh et al., 2011; Thomas et al., 2013; Swiezawska et al., 2014; Chatukuta et al., 2018). Furthermore, exogenously applied cAMP causes stomatal opening (Curvetto et al., 1994) and modulates ion transport through cyclic nucleotide gated channels (CNGCs) (Maathuis and Sanders, 2001; Balagué et al., 2003; Lemtiri-Chlieh and Berkowitz, 2004). Additionally, components of cAMP signaling pathways, as well as cAMP interacting proteins (i.e., ACs, phosphodiesterases (PDEs) and protein kinase A (PKA)) have been reported (Assmann, 1995; Gehring, 2010; Donaldson et al., 2016). To date, however, no gene encoding a PDE has been identified in higher angiosperms, although a recent study has identified a class III AC in the liverwort *Marchantia polymorpha* which also harbors a functional N-terminal PDE domain (Kasahara et al., 2016). This protein has orthologs only in basal land plants and charophytes, thus a *bona fide* PDE-AC in higher plants remains elusive. On the other hand, six ACs have now been reported in higher plants: a *Zea mays* protein that participates in polarized pollen tube growth (Moutinho et al., 2001); a *Nicotiana benthamiana* protein that plays a role in tabtoxinine-β-lactam-induced cell death during the development of wildfire disease (Ito et al., 2014); a *Hippeastrum x hybridum* HpAC1 protein (Swiezawska et al., 2014); an Arabidopsis pentatricopeptide repeat-containing protein, AtPPR (AT1G62590) (Ruzvidzo et al., 2013); an Arabidopsis Clathrin Assembly Protein (Chatukuta et al., 2018) and AtKUP7 (Al-Younis et al., 2015).

Despite the progress made in recent years regarding the structure and function of the HAK/KUP/KT family of transporters, we are still far from fully understanding the mechanism by which HAK/KUP/KT transporters operate and contribute to K^+^ homeostasis at the cellular and the whole plant level. Here we report that the N-terminal cytosolic region of AtKUP5 (AT4G33530) from *A. thaliana* contains an AC catalytic center and show that recombinant AtKUP5^1-104^ generates cAMP, detectable by enzyme immunoassay and mass spectrometry, and can rescue an *E. coli* mutant that lacks its adenylate cyclase (*cyaA*) gene, thus enabling lactose fermentation. Furthermore, we show that a functional AC center is necessary for the K^+^ transport activity of AtKUP5 since mutations that abolish AtKUP5 AC activity also prevent AtKUP5 complementation of yeast *trk1 trk2* mutants. Finally, we show that in response to K^+^, *trk1 trk2* mutants expressing AtKUP5 generate cAMP.

## Materials and Methods

### Structural Analysis of Adenylate Cyclase Centers in AtKUP5 and AtKUP5 S81P

Full-length AtKUP5 and AtKUP5 S81P 3D structures were modeled against the AtKUP7 template as provided in Al-Younis et al. (2015) using the Modeller (ver. 9.14) software (Šali and Blundell, 1993). The AtKUP5 models were visualized and analyzed, and the images were created using UCSF Chimera (ver. 1.10.1) (Pettersen et al., 2004). Docking simulations of ATP to the AC centers of AtKUP5 and AtKUP5 S81P were performed using AutoDock Vina (ver. 1.1.2) (Trott and Olson, 2010). The ATP docking poses were analyzed and docking images were created using PyMOL (ver. 1.7.4) (The PyMOL Molecular Graphics System, Schrödinger, LLC).

### Generation of Recombinant AtKUP5^1-104^

RNA was extracted from *A. thaliana* Col-0 leaf tissue using the RNeasy kit (Qiagen, Crawley, United Kingdom) and converted to cDNA using Superscript III Reverse Transcriptase according to the manufacturer’s instructions (Invitrogen, Carlsbad, CA, United States). Primers designed to amplify the AC domain of *AtKUP5* were *AtKUP5* AC forward (5′-ATGTTTCACGTGGAAGAAGAAAGC-3′) and *AtKUP5* AC reverse (5′-TCACTTTCCTATACCAGTGTCCTCG-3′). The cDNA was used as template in a PCR reaction with the *AtKUP5* AC primers and KAPA HiFi Taq Polymerase according to the manufacturer’s instructions (KAPA Biosystems, Wilmington, MA, United States). Subsequently, A overhangs were added using KAPA Taq Polymerase according to the manufacturer’s instructions (KAPA Biosystems, Wilmington, MA, United States) and the PCR product was cloned into the Gateway compatible pCR8 vector (Invitrogen, Carlsbad, CA, United States). *AtKUP5^1-104^* S81P and *AtKUP5^1-104^* S81P/D81T mutants were generated by site directed mutagenesis using the following primers: *AtKUP5* S81P forward (5′-CCGTTGACTCTTTCTATGTAGATGCTCT-3′), *AtKUP5* S81P reverse (5′-AGAGCATCTACATAGAAAGAGTCAACGG-3′), *AtKUP5* S81P/D83T forward (5′-CGTTGACCCTTTCTATGTAGATGCTC-3′) and *AtKUP5* S81P/D83T reverse (5′-GAGCATCTACATAGAAAGGGTCAACG-3′). The AC domain of *AtKUP5*, *AtKUP5^1-104^*, and the single *AtKUP5^1-104^* S81P and double *AtKUP5^1-104^* S81P/D83T mutants were recombined into the pDEST17 expression vector (Invitrogen, Carlsbad, CA, United States) to create pDEST17-*AtKUP5^1-104^* fusion constructs containing C-terminal His tags for affinity purification. These were transformed into *E. coli cyaA* mutants for functional complementation or *E. coli* BL21 A1 cells (Invitrogen, Carlsbad, CA, United States) for recombinant protein expression. Purification of the recombinant proteins was performed under denaturing conditions using Ni-NTA agarose beads according to the manufacturer’s instructions (Qiagen, Hilden, Germany) and refolded by Fast Protein Liquid Chromatography (FPLC) using HisTrap HP Ni-NTA columns (GE Healthcare, Little Chalfont, United Kingdom) as detailed elsewhere (Meier et al., 2010) and in the Supplementary Methods.

### Complementation of an Adenylate Cyclase Deficient *E. coli* Mutant

The *E. coli cyaA mutant* SP850 strain [*lam-*, *el4-*, *relA1*, *spoT1*, *cyaA1400* (*:kan*),*thi-1*] (Shah and Peterkofsky, 1991), deficient in its adenylate cyclase (*cyaA*) gene, was obtained from the *E. coli* Genetic Stock Center (Yale University, New Haven, CT, United States) (Accession Number 7200). The pDEST17-*AtKUP5^1-104^* constructs were transformed into the *E. coli cyaA* mutant strain by heat shock (2 min at 42°C). Bacteria were grown at 37°C in Luria Broth media supplemented with 100 μg/mL ampicillin and 100 μg/mL kanamycin until they reached an OD_600_ of 0.6 and then incubated with 0.5 mM isopropyl-beta-D-1-thiogalactopyranoside (IPTG) for 4 h for transgene induction prior to streaking on MacConkey agar (MacConkey, 1905).

### *In vitro* Adenylate Cyclase Enzymatic Assay and Detection of cAMP

Cyclic AMP was generated *in vitro* from reaction mixtures containing 10 μg of recombinant protein in 50 mM Tris-HCl pH 8, 2 mM isobutylmethylxanthine (IBMX; Sigma-Aldrich, St. Louis, MO, United States), 5 mM MgCl_2_ or MnCl_2_ and 1 mM ATP in a final volume of 100 μL. The cAMP produced was measured by enzyme immunoassay using the acetylation protocol in the Biotrak enzyme immunoassay kit as described by the manufacturer (GE Healthcare, Little Chalfont, United Kingdom) or by liquid chromatography tandem mass spectrometry (LC–MS/MS) on an LTQ Orbitrap Velos mass spectrometer (Thermo Fisher Scientific, Waltham, MA, United States). All methods are more extensively detailed elsewhere (Kwezi et al., 2011).

For cAMP measurements in *E. coli*, cAMP was extracted from 100 μL of culture that contains approximately 10^5^ cells according to the non-acetylation procedure for cell culture and measured by enzyme immunoassay as per the manufacturer’s instructions (GE Healthcare, Little Chalfont, United Kingdom). Cyclic AMP was measured in *Saccharomyces cerevisiae* samples essentially as described in Thevelein (1984). Briefly, 50 mg of culture was filtered using Whatman glass fiber filters. The filter papers were then transferred to 50 mL tubes that contained 2 mL glass beads and 250 μL ice cold 1 M HClO_4_. The tubes were subjected to successive rounds of agitation with a vortex for 3 min followed by freezing in liquid N_2_ to lyse the cells. The samples were centrifuged at 10,000 × *g* and the cAMP content of the supernatant determined by LC–MS/MS.

### Electrophysiology of AtKUP5

Full length *AtKUP5* was PCR amplified from *A. thaliana* Col-0 leaf cDNA using *AtKUP5* forward (5′-ATGTTTCACGTGGAAGAAGAAG-3′) and *AtKUP5* no stop reverse (3′-TACCATATAAGTCATTCCAACTTG-5′) primers and cloned into the pCR8 vector (Invitrogen, Carlsbad, CA, United States) as described above. *AtKUP5* was recombined into the Vivid Colors^TM^ pcDNA^TM^6.2/C-EmGFP-DEST vector and transformed into One Shot^®^ Mach1^TM^-T1R phage-resistant chemically competent *E. coli* cells and plasmid DNA was extracted using the Invitrogen^TM^ PureLink^®^ HQ Mini Plasmid Purification kit according to the manufacturer’s instructions (Invitrogen, Carlsbad, CA, United States).

Frozen HEK-293 cells were thawed and transferred to a T-75 cm^2^ culture flask containing 11 mL pre-warmed complete media: Dulbecco’s modified Eagle’s medium DMEM (1x), GlutaMAX^TM^-I supplemented with 10% (v/v) fetal bovine serum and 1% (v/v) penicillin-streptomycin (10,000 U/mL) (Life Technologies Europe BVM, Bleiswijk, Netherlands). The cell culture was grown at 37°C in a 5% CO_2_ humidified growth incubator and the media replaced 24 h after seeding. On the day of transfection, HEK-293 cells were grown to 90% confluency; the media was replaced with 2 mL of 0.25% (w/v) trypsin-EDTA solution (Sigma-Aldrich, St. Louis, MO, United States) and the cells were incubated for 6 min until they started to detach. Then 6 mL of complete media was added to stop the digestion. Cells were further detached by gentle pipetting after which cell viability (trypan blue) and cell counts were determined using a cell counter (Countess^®^ II Automated Cell Counter, Thermo Fisher Scientific, Waltham, MA, United States). Cells were diluted to 2.5 × 10^5^ viable cells/2 mL with pre-warmed complete medium. Lipofectamine^®^ 3000 was used to transiently transfect the HEK-293 cells with 2.5 μg *ATKUP5-EmGFP* or empty vector according to the manufacturer’s instructions (Sigma-Aldrich, St. Louis, MO, United States). The cell mixture was transferred to a 6-well culture dish containing 12 mm oval cover glasses that had been cleaned and coated with poly-D-Lysine hydrobromide (Sigma-Aldrich, St. Louis, MO, United States). The transfected cells were incubated overnight at 37°C and 5% CO_2_ to allow attachment to the cover glass.

The HEK-293 cells grown on cover glasses were transferred into a home-made recording chamber, permanently perfused with external solution at a rate of 0.5–1 mL per min. An inverted Carl Zeiss Axio Observer.A1 microscope (Carl Zeiss, Oberkochen, Germany) fitted with a green fluorescence module was used to visualize HEK-293 positively transfected with *ATKUP5-EmGFP*. Recording pipettes were pulled from thick/standard wall borosilicate glass capillaries (B150-86-10) using a P-1000 FLAMING/BROWN micropipette puller (Sutter Instrument^®^, Novato, CA, United States). The pipette resistance was 3–5 MΩ when filled with an intracellular solution consisting of 100 mM KCl, 1 mM CaCl_2_, 4 mM MgCl_2_, 10 mM EGTA, and 10 mM HEPES at pH 7.3 (Osmolality: 290 ± 5 mOsm, adjusted with sorbitol). The external bath solution contained 10 mM KCl, 1 mM CaCl_2_, 1 mM MgCl_2_, 10 mM HEPES, and 10 mM glucose at pH 7.3 (Osmolality: 310 ± 5 mOsm, adjusted with sorbitol). Upon achieving whole-cell configuration, the cells were maintained at -60 mV, a holding potential close to the Nernstian potential equilibrium for K^+^ (resting membrane was governed by the concentrations of K^+^ used in and out of the cell). A simple gap-free protocol was used to record the running background current generated at this holding potential and monitor any specific currents derived from the activity of *AtKUP5-EmGFP* when the external pH was rapidly switched from 7.3 to 5.3. As a control, we compared these currents to those in HEK-293 cells expressing the empty vector. We also tested other voltages between +120 and -80 mV using protocols consisting of a series of 1 s long squared voltage depolarization. Pulse protocols, data acquisition and analysis were performed using a MultiClamp^TM^ 700B microelectrode amplifier and pClamp ver. 10 software package (Axon Instruments, Inc., Sunnyvale, CA, United States). All signals were low-pass filtered at 2 kHz before analog-to-digital conversion and were uncorrected for leakage current or capacitive transients.

### Complementation of K^+^ Transport Deficient *S. cerevisiae* Mutant

Full length *AtKUP5* was PCR amplified from *A. thaliana* Col-0 leaf cDNA using the *AtKUP5* forward and *AtKUP5* reverse (3′-TCATACCATATAAGTCATTCCAACTTG-5′) primers and cloned into the pCR8 vector (Invitrogen, Carlsbad, CA, United States) as described above. Single *AtKUP5* S81P and double *AtKUP5* S81P/D83T mutations were generated by site directed mutagenesis as described above. The full length *AtKUP5*, *AtKUP5* S81P and *AtKUP5* S81P/D83T constructs were then recombined into the yeast expression vector, pYES-DEST52 (Invitrogen, Carlsbad, CA, United States).

The *S. cerevisiae trk1 trk2* mutant strain (CY162) was kindly provided by Richard Gaber (Northwestern University, Evanston, IL, United States). Competent cells were generated using the *S. c.* EasyComp transformation kit and transformed with the pYES-DEST52-*AtKUP5* constructs according to the manufacturer’s instructions (Invitrogen, Carlsbad, CA, United States). Briefly, either 5 μg of pYES-DEST52 (empty vector) or pYES-DES52-*AtKUP5* were transformed into competent *trk1 trk2* yeast and 100 μL of the transformation reaction was plated onto uracil-deficient selective media (SC-ura) supplemented with 100 mM KCl. The empty vector or pYES-DEST52-*AtKUP5* transformants were tested for their ability to complement the yeast *trk1 trk2* mutant as follows: a single colony was inoculated into 15 mL SC-ura supplemented with 2% (w/v) raffinose, 2% (w/v) glucose and 100 mM KCl and grown overnight at 30°C with shaking. The next day the OD_600_ was recorded and the culture volume required to obtain an OD_600_ of 0.4 in 50 mL was calculated. This volume of overnight culture was centrifuged at 1500 × *g* for 5 min at 4°C and the cell pellet washed in 1–2 mL distilled water. The centrifugation step was repeated, and the cells were resuspended and incubated in 50 mL induction medium [SC-ura supplemented with 2% (w/v) raffinose, 2% (w/v) galactose and 100 mM KCl] at 30°C with shaking for 8 h to induce *AtKUP5* expression. A serial dilution was performed and 5 μL of each dilution was plated onto induction medium containing either 0.02 mM KCl or 20 mM KCl and incubated for 2–3 days at 30°C.

### K^+^ Measurement

K^+^ was measured in *S. cerevisiae trk1 trk2* mutants transformed with empty vector, full length *AtKUP5*, *AtKUP5* S81P, or *AtKUP5* S81P/D83T. The cells were grown in 50 mL induction medium containing 0.02 mM KCl for 8 h at 30°C with shaking to induce *AtKUP5* expression, as described above. The cell pellets were collected by centrifugation and washed three times in 10 mL water. The pellets were then dried and weighed. For analysis of K^+^ content by inductively coupled plasma optical emission spectrometry (ICP-OES), the samples were digested using acid microwave assisted digestion. Briefly, 5 mL HNO_3_ was added and the samples were incubated in a 1100 W microwave at 200°C for 10 min followed by 220°C for 20 min. Thereafter, the samples were diluted to 25 mL with water. Once the samples had cooled, K^+^ was measured using an ICP-OES Varian 720-ES spectrometer (Varian, Palo Alto, CA, United States) calibrated with the single-element standard for K^+^ (Inorganic Ventures, Christiansburg, VA, United States).

## Results

### Discovering Arabidopsis Candidate Adenylate Cyclases

**FIGURE 1:**
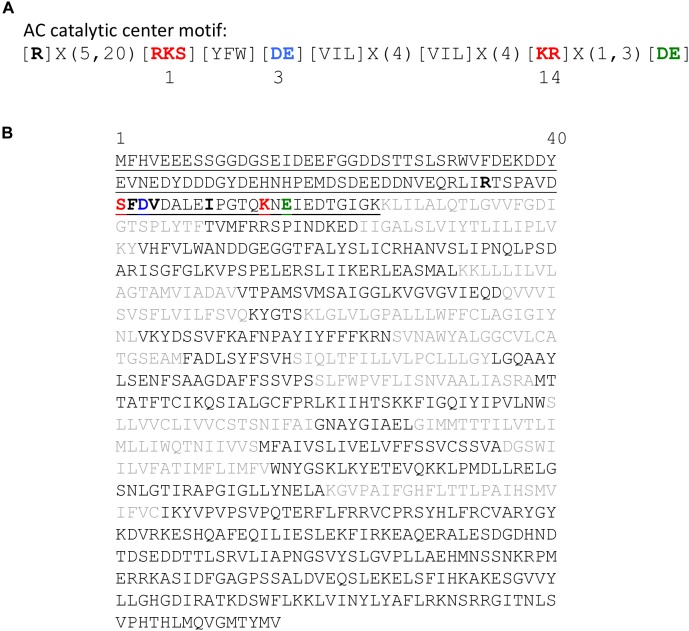
Features of the adenylate cyclase catalytic center of the AtKUP5 K^+^ transporter. **(A)** The 14 amino acid motif of annotated and experimentally tested AC catalytic centers. **(B)** The amino acid sequence of the AtKUP5 K^+^ transporter. The AC catalytic center is located in the cytosolic N terminal region and is shown in bold and the 104 amino acid fragment tested for AC activity is underlined. The 12 transmembrane domains are shown in gray font.

We have applied a 14 amino acid core search term derived from functionally assigned residues in the catalytic center of guanylyl cyclases (GCs) (Tucker et al., 1998; Ludidi and Gehring, 2003; Wong and Gehring, 2013) and adenylate cyclases (ACs) (Tucker et al., 1998; Roelofs et al., 2001; Gehring, 2010; Wong and Gehring, 2013) to identify candidate nucleotide cyclases, and in particular ACs, in the model plant, *A. thaliana*. The AC search motif used in this study (Figure 1A) comprises the residue that performs the hydrogen bonding with guanine or adenine [RKS] in position 1, and the amino acid that confers substrate specificity, [CTGH] for GCs or [DE] for ACs, in position 3. The amino acid in position 14, [KR] stabilizes the transition state from GTP or ATP to cGMP or cAMP, respectively, and the [DE] at 1–3 residues downstream from position 14, participates in Mg^2+^/Mn^2+^-binding (Tucker et al., 1998). The core motif also contains the amino acids with hydrophobic side chains [YFW] situated between the assigned residue that performs the hydrogen bonding (position 1) and the amino acid that confers substrate specificity (position 3) (Figure 1A). This core AC motif ([RKS][YFW][DE][VIL]X(8,9)[KR]X(1,3)[DE]) retrieves 341 Arabidopsis proteins which seems to be a large number of hits that may contain false positives. To increase stringency, we used additional filters in the form of a hydrophobic residue [VIL], typical for experimentally tested plant GCs (Ludidi and Gehring, 2003; Kwezi et al., 2007, 2011; Meier et al., 2010; Qi et al., 2010), in position 9 and a pyrophosphate binding [R] (Liu et al., 1997) 5–20 amino acids upstream of position 1 which is known to be involved in pyrophosphate binding in class III but not class II GCs (Roelofs et al., 2001). The core motif with the additional filters retrieved a narrower list of hits (14 in total) and importantly, identified candidate AC proteins of higher confidence (Supplementary Table 1), one of which is AtKUP5 (AT4G33530). This protein is predicted to harbor 12 transmembrane domains, typical of the HAK/KUP/KTs (Gierth and Maser, 2007), with the AC core catalytic center located in the N-terminal cytosolic domain (Figure 1B) spanning amino acids 81 to 96.

### Structural Analysis of the AtKUP5 Adenylate Cyclase Center

**FIGURE 2:**
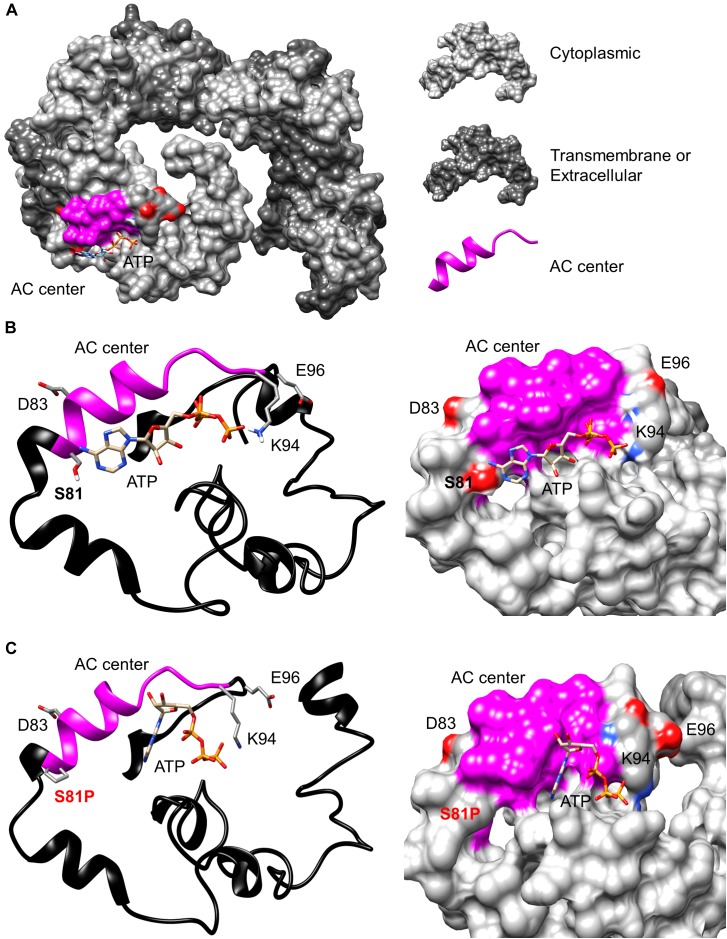
Modeling of the adenylate cyclase catalytic center of AtKUP5. **(A)** The full-length AtKUP5 model showing the location of the AC center at the solvent-exposed cytosolic region. Dockings of ATP and the interactions of ATP with the key residues at the AC catalytic centers of **(B)** wild type AtKUP5 and **(C)**, AtKUP5 S81P are shown as ribbon and as surface models in the left and right panels, respectively. The AC center is highlighted in pink and the amino acid residues implicated in interactions with ATP and the metal-binding residue (E96) are colored according to their charges (red being negative and blue being positive) in the surface models and shown as individual atoms in the ribbon model. The full-length AtKUP5 and AtKUP5 S81P 3D structures were modeled against the AtKUP7 template using the Modeller (ver. 9.14) software. ATP docking simulations were performed using AutoDock Vina (ver. 1.1.2) and images were created using PyMOL (ver. 1.7.4).

In addition to the identification of a candidate AC catalytic center in AtKUP5 using a rationally designed AC motif, we also assessed, using computational methods, the feasibility that the AC center can bind the substrate, ATP and catalyze its subsequent conversion to cAMP. Since AtKUP5 has 41% amino acid identity to AtKUP7, we modeled AtKUP5 against an AtKUP7 structure described previously (Al-Younis et al., 2015). We show in this model that the AC catalytic center is solvent exposed thus allowing for unimpeded substrate interactions and presumably also for catalysis (Figure 2A). Further probing of the AC center by molecular docking of ATP suggests that ATP can dock at the AC center with a good free energy (-5.9 kcal/mol) and a favorable binding pose. Specifically, the negatively charged phosphate end of ATP points toward Lys94 (position 14 of the AC motif) while the adenosine end is surrounded by negatively charged residues provided by Ser81 (position 1 of the AC motif) (Figure 2B) much like in the structurally resolved GC centers (Wheeler et al., 2017). Consistently, when Ser81 was mutated to Pro81, docking simulations indicate that the favorable ATP pose (shown in Figure 2B) was abolished (Figure 2C). The adenosine end of ATP now assumes a configuration that is too distant for any interactions with Pro81 and this is presumably due to the loss of negative charges together with the ability to form hydrogen bonds with ATP. This suggests that the AC catalytic activity of an AtKUP5 S81P mutant may be impaired.

### AtKUP5^1-104^ Rescues an Adenylate Cyclase Deficient *E. coli* Mutant

**FIGURE 3:**
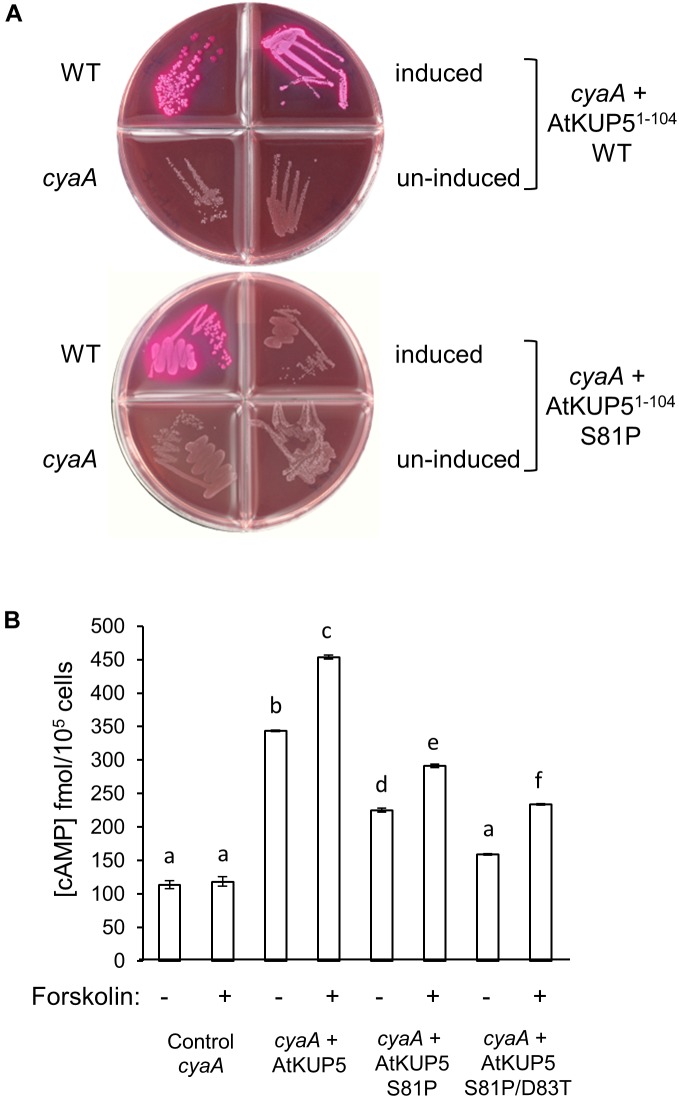
Functional complementation of an AC deficient *Escherichia coli* mutant with AtKUP5^1-104^. **(A)** The AtKUP5 AC domain (AtKUP5^1-104^) complements the *E. coli cyaA* mutant (SP850) as indicated by the growth of red colonies on MacConkey agar (upper panel). The mutated AC domain, AtKUP5^1-104^ S81P, was unable to rescue the *cyaA* mutant (lower panel). **(B)** Total cAMP measured by enzyme immunoassay in *cyaA* bacterial extracts treated with 0.5 mM IPTG for 4 h to induce expression of AtKUP5^1-104^, AtKUP5^1-104^ S81P or AtKUP5^1-104^ S81P/D83T, with or without 100 μM forskolin. Data are the mean values for three independent experiments (*n* = 3) and error bars are standard errors of the mean. Statistical analysis was performed by one-way ANOVA followed by a Tukey–Kramer multiple comparison test. Different letters indicate data that are significantly different from each other. The *E. coli cyaA* mutant expressing AtKUP5^1-104^ produced significantly more cAMP than untransformed cells and this activity was stimulated by forskolin. Mutations in the AtKUP5 AC domain significantly reduced the cAMP in the bacterial extracts when compared to the wild type AtKUP5^1-104^.

To investigate if the designated AC domain of AtKUP5 can function as an AC, we tested its ability to rescue an AC deficient *E. coli* mutant. AtKUP5^1-104^ was cloned and expressed in an *E. coli* SP850 strain that lacks its endogenous AC gene (*cyaA*) that in turn prevents lactose fermentation. As a result of the *cyaA* mutation, the AC-deficient *E. coli* and the un-induced transformed *E. coli* remain colorless when grown on MacConkey agar (Figure 3A). In contrast, the AtKUP5^1-104^ transformed *E. coli* SP850 cells, when induced with 0.5 mM IPTG, form red colored colonies much like the wild type *E. coli* (Figure 3A, upper panel), indicating that a functional AC center in the recombinant AtKUP5^1-104^ has rescued the *E. coli cyaA* mutant growing on MacConkey agar. When the Ser81 in position 1 of the AC motif (that hydrogen bonds with ATP) is mutated to a Pro81, the mutated AtKUP5 AC can no longer rescue the *E. coli cyaA* mutant (Figure 3A, lower panel). Similarly, full length AtKUP5 was able to rescue the *E. coli cyaA* mutant but full length AtKUP5 S81P could not (Supplementary Figure 1A). This suggests that the S81P mutation in the AtKUP5 AC domain has abolished the AC activity. This is supported by cAMP measurements that show that expression of AtKUP5^1-104^ in the *E. coli cyaA* mutant dramatically increases cAMP levels (Figure 3B). Moreover, this activity is stimulated by forskolin, a typical activator of ACs (Seamon and Daly, 1981). Significantly, the AtKUP5^1-104^ S81P mutant has much lower cAMP levels than the wild type AtKUP5^1-104^ and this is also observed in a double mutant AtKUP5^1-104^ S81P/D83T which is additionally modified at the residue hypothesized to be involved in substrate specificity. The double mutant is also unable to rescue the *E. coli cyaA* mutant (Supplementary Figure 1B). These data suggest that AtKUP5^1-104^ contains a functional AC that can complement the *E. coli cyaA* mutant and that Ser81, as predicted, is critical to the AC activity of AtKUP5.

### *In vitro* Adenylate Cyclase Activity of Recombinant AtKUP5^1-104^

**FIGURE 4:**
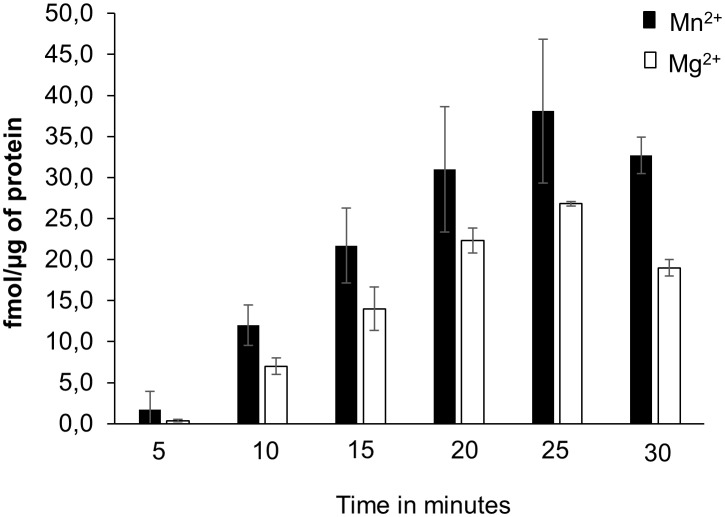
cAMP generated by recombinant AtKUP5^1-104^. cAMP was generated from a reaction mixture containing 10 μg of the purified recombinant AtKUP5^1-104^, 50 mM Tris-HCl pH 8; 2 mM IBMX, 1 mM ATP and either 5 mM MnCl_2_ or MgCl_2_ as a cofactor, at different time points, as measured by enzyme immunoassay.

**FIGURE 5:**
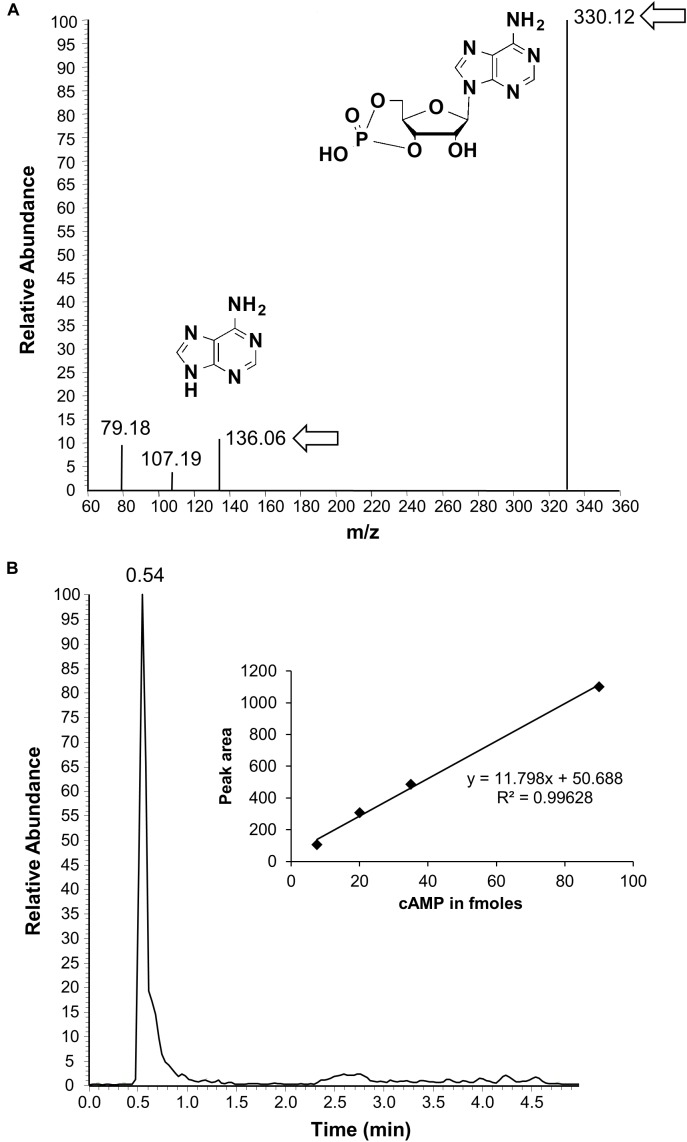
Detection of cAMP generated by AtKUP5^1-104^ by liquid chromatography tandem mass spectrometry. **(A)** Is a representative ion chromatogram of cAMP showing the parent and daughter ion peaks (see arrows and inset for the structures) and **(B)** is an HPLC elution profile of cAMP with a calibration curve shown in the inset. The calculated amount of cAMP generated from a reaction mixture containing 10 μg of the purified recombinant AtKUP5^1-104^, 50 mM Tris-HCl pH 8; 2 mM IBMX, 5 mM MnCl_2_, and 1 mM ATP after 25 min is 49 fmol/μg protein.

To test if the AtKUP5 AC center generates cAMP *in vitro*, AtKUP5^1-104^ was recombinantly expressed in *E. coli* and affinity purified (Supplementary Figure 2). The AC activity of AtKUP5^1-104^ was tested in a reaction mixture containing ATP and either Mg^2+^ or Mn^2+^ as the cofactor, since mammalian ACs can be activated by either divalent cation (Hurley, 1999), and the cAMP generated was measured by enzyme immunoassay. Significantly, cAMP accumulated after the reaction had occurred for 10 min and continued to accumulate until a maximum activity was reached after 25 min, generating 38.1 fmol cAMP/μg protein in the presence of Mn^2+^ and 26.8 fmol cAMP/μg protein in the presence of Mg^2+^ (Figure 4). Cyclic AMP levels were also measured by LC–MS/MS specifically identifying the presence of the unique product ion at m/z 136 [M + H]^+^ that is fragmented in the second ionization step, in addition to the parent ion at m/z 330 [M + H]^+^ (Figure 5A). This fragmented product ion was used for quantitation (Figure 5B). In the presence of Mn^2+^, the recombinant AtKUP5^1-104^ generates cAMP that increases with time achieving a maximum amount of 49 fmol cAMP/μg protein at 25 min. Therefore, the designated AtKUP5 AC center can generate cAMP *in vitro* as measured by both enzyme immunoassay and LC–MS/MS and has a preference for Mn^2+^ as a co-factor.

### Electrophysiology of AtKUP5

**FIGURE 6:**
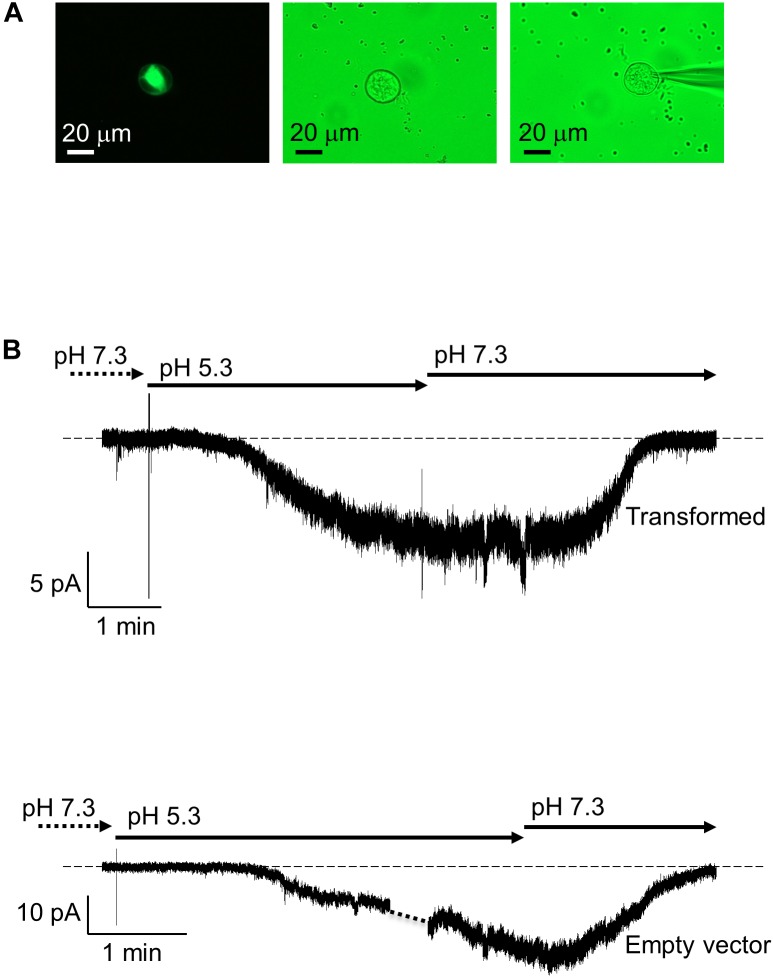
Patch clamp recordings of AtKUP5 in HEK293 cells. **(A)** Fluorescent (left panel) and bright field (middle and right panels) images of a HEK293 cell transiently transfected with *AtKUP5-EmGFP* and shown with the patch pipette (right panel) viewed at 40x magnification. **(B)** Whole-cell patch clamp recordings of HEK293 cells transformed with *AtKUP5-EmGFP* (upper panel) or empty vector (lower panel). In both conditions, a similar inward current in size (approximately –8 to –12 pA) is activated in a reversible manner when external pH is switched from 7.3 to 5.3. The dashed line corresponds to zero electric of the amplifier.

To investigate whether AtKUP5 transports K^+^, full length *AtKUP5* was transfected into HEK-293 cells (Figure 6A) and its ability to generate currents was measured by whole-cell patch clamp. When the holding membrane voltage (*V*_h_) was set to -60 mV with the external solution at pH 7.3, all HEK-293 cells transfected with *AtKUP5* (*n* = 4) had running background current close to 0 (ranging between -3 and +1 pA). Upon switching to pH 5.3, an inwardly-directed current developed steadily over a period of 2–3 min to reach a steady-state ranging between -8 and -12 pA. A representative experiment is shown (Figure 6B, upper panel). A similar effect was also observed in HEK-293 cells transfected with the empty vector (Figure 6B, lower panel). This effect was fully reversible upon washout in both sets of HEK-293 cells. The data seem to indicate that both non-expressing and expressing-*AtKUP5* HEK-293 cells have an intrinsic transport mechanism activated by protons and that AtKUP5 is not contributing to this since no additional sizable current is seen in HEK-293 cells expressing *AtKUP5*. Attempts were also made to see if HEK-293 cells expressing *AtKUP5* show any novel/unusual currents at other depolarized voltages (from +120 to -80 mV in -20 mV decrements) but they did not (data not shown). Mostly, at these depolarized voltages and using our internal and external media (see section “Materials and Methods”), we only recorded the common intrinsic I_A_-like K^+^-current, arising from a channel native to HEK-293 cells (Ooi et al., 2016).

### AtKUP5 rescues a K^+^ Transport Deficient *S. cerevisiae* Mutant

**FIGURE 7:**
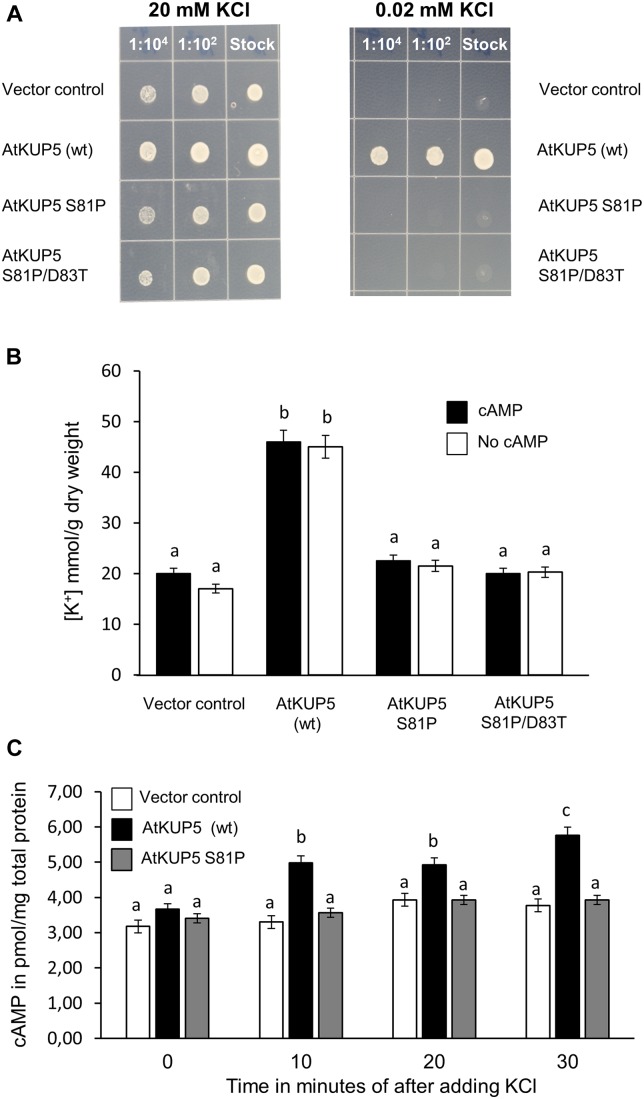
Functional complementation of K^+^ transport deficient *Saccharomyces cerevisiae* mutant with AtKUP5. *S. cerevisiae trk1 trk2* mutants were transformed with full length AtKUP5, AtKUP5 S81P or AtKUP5 S81P/D83T and recombinant protein expression induced by growth in inductive media (SC-ura with 2% (w/v) galactose and 2% (w/v) raffinose) for 8 h. **(A)** Induced cultures were plated onto inductive media supplemented with either 20 or 0.02 mM KCl. **(B)** K^+^ uptake was measured by ICP-OES in *trk1 trk2* transformants grown in inductive media supplemented with 0.02 mM KCl either with or without 100 mM dibutyryl cAMP. **(C)** cAMP was measured by LC–MS/MS in cultures grown in inductive media supplemented with 0.02 mM KCl and treated with 3.33 mM KCl for the indicated times. All samples were performed in triplicates with error bars representing the standard error of the mean (SEM). Statistical analysis was performed by one-way ANOVA followed by a Tukey–Kramer multiple comparison test. Different letters indicate significantly different data.

Since it is possible that *AtKUP5* is not functionally expressed in HEK-293 cells, the ability of AtKUP5 to functionally complement the *S. cerevisiae* K^+^ transport deficient mutant, *trk1 trk2* was interrogated. The *trk1 trk2* mutant was transformed with full length *AtKUP5* or full length *AtKUP5* containing the S81P single mutation or the S81P/D83T double mutation (both of which abolish the AC activity of AtKUP5) and compared to the empty vector control. All yeast transformants grew on 20 mM K^+^ replete media, under which conditions high affinity K^+^ transport is suppressed (Figure 7A, left hand panel). Notably, under 0.02 mM K^+^ deficient conditions, wild type AtKUP5 could functionally complement the *trk1 trk2* mutant that is impaired in its high affinity K^+^ transport systems; however, the AtKUP5 mutants that lack a functional AC were unable to complement the *S. cerevisiae* mutant (Figure 7A, right hand panel). This suggests that AtKUP5 functions as a K^+^ transporter *in vivo* and that its functionality as a K^+^ transporter is dependent on the presence of a functional AC that can generate cAMP from ATP.

The relationship between the K^+^ transport activity and the AC activity of AtKUP5 was thus investigated further. To test whether cAMP could rescue the deficiency in K^+^ transport observed in the AtKUP5 S81P and AtKUP5 S81P/D83T mutants, the various yeast transformants were grown in K^+^ deficient media with or without the cell permeable dibutyryl-cAMP and the amount of K^+^ taken up was measured by ICP-OES. As expected, the *trk1 trk2* mutant yeast transformed with the full length, wild type AtKUP5 accumulated significantly more K^+^ compared to the *trk1 trk2* mutant transformed with the empty vector or either of the full length AtKUP5 constructs with the mutated AC domains (Figure 7B). However, treatment with cAMP did not affect the amount of K^+^ accumulated in any of the yeast transformants.

Conversely, to test whether K^+^ has any effect on the AC activity of AtKUP5, cAMP levels were measured in the *trk1 trk2* mutant transformed with either empty vector, full length wild type AtKUP5 or AtKUP5 S81P and grown in 0.02 mM K^+^ supplemented media, then treated with 3.33 mM K^+^. The K^+^ treatment significantly increased cAMP levels in the *trk1 trk2* mutant transformed with wild type AtKUP5 10 min following treatment and this effect was sustained for at least 30 min (Figure 7C). There was no increase in cAMP levels following K^+^ treatment in the *trk1 trk2* mutant transformed with the empty vector or AtKUP5 S81P. This suggests that transport of K^+^ via AtKUP5 generates cAMP and that it is the AC domain of AtKUP5 that is responsible for the generation of cAMP in the transformed yeast in response to K^+^ treatment. The absence of a similar increase in cAMP levels in the AtKUP5 S81P mutant further validates our previous findings that Ser81 is critical for the AC activity of AtKUP5.

## Discussion

In canonical GCs and ACs, the catalytic site is formed by two separate protein chains, which dimerize to form the active site (Potter, 2011). Unlike canonical GCs and ACs, plant GCs and ACs do not contain the full cyclase domains but their catalytic centers are often found embedded within complex proteins of other primary functions (Kwezi et al., 2007). These plant proteins exist as multi-domain complexes that accommodate functional GC or AC centers at moonlighting sites (Wong et al., 2015). Therefore, the overall structure is entirely different from canonical GCs or ACs as they assume folds that reflect their primary functions, such as acting as receptors or protein kinases. For this reason, our AC search motif was built to include conserved residues at catalytic sites of canonical GCs or ACs regardless of which chain of the dimers they may have come from and this motif has been subjected to a series of rational modifications according to methods detailed in Chatukuta et al. (2018) (Figure 1A). Using this AC search motif, we have identified a list of 14 candidate ACs in *A. thaliana*, one of which is the K^+^-uptake transporter, AtKUP5 (Supplementary Table 1 and Figure 1B). Included in this list is the closely related AtKUP7 which we have previously shown to be a functional AC (Al-Younis et al., 2015).

The structure of AtKUP5 is very similar to that of AtKUP7. Structural analysis and docking simulations suggest that the catalytic center of AtKUP5 is solvent exposed and can bind ATP through interactions with key residues in the AC motif supporting that AtKUP5 could function as an AC and that the conserved residues in the motif are functionally important (Figures 2A,B). Importantly, we have shown that the AC domain of AtKUP5 can both complement an *E. coli* mutant that lacks its endogenous AC gene, *cyaA* (Figure 3A, upper panel) and produce cAMP in this mutant (Figure 3B) as well as *in vitro* (Figure 4). We note that the AC activity of the recombinant AtKUP5 AC domain is 10–50 times lower than the animal ACs, however is similar to the activity reported for other plant ACs and GCs (Kwezi et al., 2007, 2011; Al-Younis et al., 2015). The lower activity of plant ACs when compared to animal ACs may be due to the localized micro-regulatory role of plant AC centers that act as rapid molecular switches capable of shifting from one signaling network to another much like plant GCs, e.g., the phytosulfokine receptor, PSKR1 (Muleya et al., 2014) and the brassinosteroid receptor, AtBRI1 (Wheeler et al., 2017).

The computational analysis also predicted that mutation of a key residue in the AC motif (position 1 that performs the hydrogen binding with ATP) from Ser81 to Pro81, would prevent ATP binding and thus impair the AC activity of AtKUP5 (Figure 2C). In agreement with this, when we mutated Ser81 to Pro81, the AtKUP5 AC domain could no longer complement the *E. coli cyaA* mutant (Figure 3A, lower panel) nor improve its cAMP levels (Figure 3B). Altogether this provides computational and experimental evidence that AtKUP5 can function as an AC and that Ser81 is critical to the AC activity of AtKUP5.

This prompted us to question the functional relevance of the AtKUP5 AC domain. To examine the function of AtKUP5 as a K^+^ transporter, we expressed AtKUP5 in HEK-293 cells; however, we failed to detect any additional current in HEK-293 cells expressing *AtKUP5* (Figures 6A,B, upper panel) when compared to HEK-293 cells transformed with empty vector (Figure 6B, lower panel). One explanation could be that the HEK-293 cells are indeed expressing a functional transporter but it is not generating a net electric charge (i.e., the K^+^-transport mechanism of AtKUP5 is not an electrogenic one). However, another possible explanation could be that, in HEK-293 cells, *AtKUP5* is simply not functionally expressed. While the inability of HAK/KUP/KT transporters to generate a current in heterologous systems such as *Xenopus* oocytes has been documented (Kim et al., 1998; Scherzer et al., 2015), HAK/KUP/KT transporters have been shown to functionally complement *E. coli* and *S. cerevisiae* mutants that are impaired in their ability to uptake K^+^ (Ahn et al., 2004). This inconsistency could be explained by the fact that signaling components required for activation of HAK/KUP/KTs are missing from the heterologous animal systems but are present in bacterial and yeast systems. In support of this, Scherzer et al. (2015) showed that DmHAK5 from Venus flytrap required co-expression of the calcineurin B-like protein 9 (CBL9) and the CBL-interacting kinase, CIPK23 to generate K^+^ currents in *Xenopus* oocytes. It is thus interesting to note that HAK/KUP/KT are found throughout bacteria, fungi and plants but are missing from the protist and animal lineage (Grabov, 2007).

Indeed, full length AtKUP5 could rescue a *S. cerevisiae trk1 trk2* mutant that is deficient in high affinity K^+^ uptake (Figure 7A) suggesting that AtKUP5 does function as a K^+^ transporter *in vivo*. Unexpectedly, the AtKUP5 mutants that lack AC activity, AtKUP5 S81P and AtKUP5 S81P/D83T, were unable to complement the *S. cerevisiae trk1 trk2* mutant (Figure 7A). This was surprising because the AtKUP5 AC domain is located in the N-terminal cytoplasmic tail and so we did not expect these mutations to affect the transmembrane pore forming region of AtKUP5. Instead this could suggest that the K^+^ transport activity of AtKUP5 is somehow linked to its AC activity.

One possibility is that cAMP generated by AtKUP5 can directly act on the channel to modulate its own K^+^ transport activity, for example through gating. Indeed, cyclic nucleotides have been previously shown to modulate the activity of K^+^ channels (Li et al., 1994; Hoshi, 1995; Leng et al., 1999). In such a case one might expect that the application of membrane permeable cAMP could rescue the deficiency in K^+^ transport observed in the AtKUP5 S81P and AtKUP5 S81P/D83T mutants. However, we found that dibutyryl-cAMP did not have any effect on any of the yeast strains (Figure 7B). It seems unlikely that this is due to a permeability issue since others have used dibutyryl-cAMP in *trk1 trk2* yeast with effect (Mercier et al., 2004) and we have observed the same result with another membrane permeable cAMP analog, 8-Br-cAMP (data not shown).

More likely, the N-terminal cytoplasmic tail of AtKUP5 regulates K^+^ transport activity possibly by mediating dimerization, protein–protein interactions and/or through phosphorylation. Thus, the S81P mutation not only abolishes the AC activity but also regulatory sites which may be necessary for K^+^ transport activity. As a result, K^+^ transport which was shown to be dependent on a functional AC center, is also affected. We note that from our model, Ser81 is both cytoplasmic and solvent exposed thus allowing for these regulatory roles (Figure 2A). Dimerization of K^+^ channels has been reported however not for the HAK/KUP/KT family of K^+^ transporters (Rocchetti et al., 2012; Shabala et al., 2016). Interestingly though, the cytosolic domain of AtPSKR1, that includes its GC center, has been shown to reversibly dimerize in solution (Muleya et al., 2016). Consistent with our hypothesis, phosphorylation of AtKUP5 at Ser81 has previously been reported Supplementary Table 1 in Xue et al. (2013) and Supplementary Table 2 in Zhang et al. (2013). Given that Ser81 has been identified as a key residue within the AC center, a change in phosphorylation state of this amino acid could not only activate the AC center but also modulate the K^+^ transport activity of AtKUP5. We note that one other amino acid within the AC center, Tyr92 and two amino acids upstream of the motif (Thr75 and Ser76) have also been found to be phosphorylated (Reiland et al., 2011; Mattei et al., 2016). Such a high density of phosphorylation sites in or within the proximity of the AC center may indicate activation of catalytic activity and/or prime protein–protein interactions. Taken together, our results indicate that Ser81 is necessary for both AC activity and K^+^ transport in AtKUP5 probably due to its involvement in phosphorylation of AtKUP5.

As discussed above, mutation of the AtKUP5 AC domain could affect K^+^ transport activity through a mechanism that is not directly linked to cAMP. Nevertheless, there is certainly a link between the K^+^ transport and AC activities of AtKUP5. We have shown that treatment of the *AtKUP5* expressing *trk1 trk2* with KCl stimulates cAMP production suggesting that K^+^ uptake via AtKUP5 can stimulate AC activity (Figure 7C). Moreover, KCl does not simulate cAMP production in AtKUP5 S81P mutant supporting that cAMP production in *trk1 trk2* in response to KCl is via the AtKUP5 AC domain (Figure 7C). Interestingly, in *Paramecium* cAMP formation was reported to be stimulated by K^+^ conductance; and this conductance in turn is an intrinsic property of the AC. The *Paramecium* multi-domain protein acts as both an AC and a K^+^ channel where a canonical S4 voltage-sensor occupies the N-terminal and a K^+^ pore-loop sits in the C-terminus on the cytoplasmic side (Weber et al., 2004). Incidentally, AtKUP5 also has such dual domain architecture as characterized by its K^+^ transport activity and its cytosolic N-terminal AC center.

**FIGURE 8:**
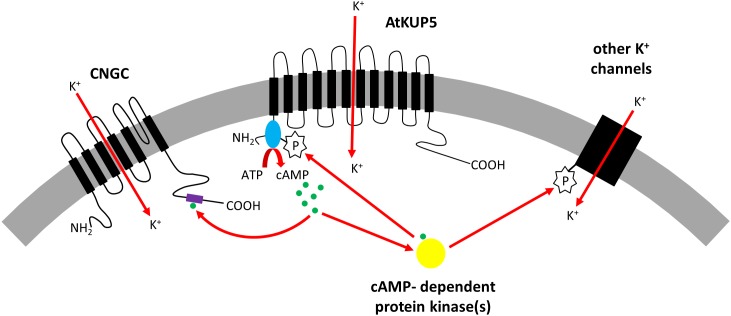
Model showing proposed role for AtKUP5 in K^+^ homeostasis. AtKUP5 transports K^+^ into the cell and generates cAMP (shown in green) through its cytosolic N-terminal adenylate cyclase domain (shown in blue). This cAMP could bind to the cyclic nucleotide binding domain (shown in purple) of cyclic nucleotide gated channels (CNGCs) which can also transport K^+^ into the cell. Additionally, cAMP could bind to cAMP-dependent protein kinases (shown in yellow) which could phosphorylate other K^+^ channels or AtKUP5, possibly at Ser81 in the adenylate cyclase domain, to further modulate K^+^ homeostasis.

In summary, we report the identification of an AC catalytic center in the N terminal cytosolic domain of AtKUP5^1-104^ discovered using rationally curated motif-based searches and supported by computational simulation of AtKUP5 models, and we show that AtKUP5^1-104^ generates cAMP *in vitro* and complements an AC deficient *E. coli* mutant, *cyaA*. Docking simulations predict that mutation of Ser81 in the AC domain prevents ATP binding to the catalytic center and AtKUP5^1-104^ S81P mutants do not generate cAMP *in vitro* or complement *E. coli cyaA* suggesting that Ser81 is required for AtKUP5 AC activity. We have also demonstrated that AtKUP5 is a K^+^ transporter as it can functionally complement a K^+^ transport deficient *S. cerevisiae trk1 trk2* mutant and increases K^+^ levels in these transformants. The S81P mutation in the AtKUP5 AC domain abolishes the functional complementation of the *S. cerevisiae trk1 trk2* mutant probably because phosphorylation of Ser81 regulates the K^+^ transport activity of AtKUP5. Interestingly though, treatment of the *S. cerevisiae trk1 trk2* expressing AtKUP5 with KCl stimulates cAMP production suggesting that K^+^ uptake via AtKUP5 can stimulate AC activity. Therefore, we propose that AtKUP5 may operate as a cAMP-dependent K^+^ flux sensor. Increases in cAMP levels in response to K^+^ transport activity of AtKUP5 could initiate downstream signal transduction cascades that act on CNGCs or protein kinases that in turn could phosphorylate AtKUP5 to fine tune K^+^ homeostasis *in planta* (Figure 8).

## Author Contributions

CG and LD conceived of the project. LD and IA-Y planned the experiments. IA-Y performed the molecular biology and cell biology experiments. FL-C did the electrophysiology. AW did the structure modeling. All authors contributed to the data analyses, the drafting and revising of the manuscript.

## Conflict of Interest Statement

The authors declare that the research was conducted in the absence of any commercial or financial relationships that could be construed as a potential conflict of interest.
