# Supplementary material for: The Arabidopsis thaliana K+-Uptake Permease 5 (AtKUP5) Contains a Functional Cytosolic Adenylate Cyclase Essential for K+ Transport
Source: Front Plant Sci. 2018 Nov 13;9:1645. doi: 10.3389/fpls.2018.01645 (PMC6243130; doi:10.3389/fpls.2018.01645)
Supplement: Supplementary file 1 [file Table_1.DOCX]

**Supplementary Methods**

**Purification and refolding of the AtKUP5^1-104^ AC domain**

The recombinant cDNA encoding *AtKUP5^1-104^* in the appropriate pDEST17-*AtKUP5^1-104^* fusion construct was transformed into BL21 A1 *E. coli* cells (Invitrogen, Carlsbad, USA) and grown in LB broth media containing 100 μg/mL ampicillin on an orbital shaker (New Brunswick Scientific, New Jersey, USA) at 200 rpm at 37°C, until the optical density (OD_600_) reached 0.6. Recombinant protein expression was induced by adding 0.2 % l-arabinose and the culture grown for a further 4 hrs at 37°C. The recombinant protein was purified by preparing a cleared cell lysate under denaturing conditions essentially as described in Protocols 10 and 17 of the QIAexpressionist manual (Qiagen, Crawley, UK) but with some modifications. Firstly, a cleared cell lysate was prepared by resuspending the harvested cells in lysis buffer (100 mM NaH_2_PO_4_, 10 mM Tris-Cl, 6 M guanidine hydrochloride; pH 8) at a ratio of 1 g pellet weight to 10 mL buffer volume and mixed with on a rotary mixer for 30 min and then centrifuged at 2300 x *g* for 15 min at room temperature. The cleared cell lysate supernatant was collected and mixed with 1 mL 50% (w/v) Ni-NTA slurry (Qiagen, Crawley, UK) that had been pre-equilibrated with 10 mL of lysis buffer. The contents were gently mixed on a rotary mixer (Breda Scientific, Breda, Netherlands) for 30 min at room temperature. The lysate-resin mixture was loaded into an empty PD-10 column (Amersham Pharmacia Biotech, Little Chalfont, UK), allowed to settle and the flow through discarded. The protein bound resin was washed three times with 30 mL wash buffer (8 M urea, 100 mM NaH_2_PO_4_, 10 mM Tris-HCl; pH 6.3) then with 2 mL elution buffer (8 M urea, 100 mM NaH_2_PO_4_, 10 mM Tris-HCl; pH 5.9) and fractions collected. The recombinant protein was subjected to a second elution with 2 mL imidazole-containing elution buffer (8 M urea, 100 mM NaH_2_PO_4_, 250 mM imidazole, 10 mM Tris-HCl; pH 8). The elution fractions that contained protein were pooled then desalted and concentrated to approximately 0.5 mL using the Amicon Ultra 15 Centrifugal Filter Unit, 15 kDa NMWL according to the manufacturer’s instructions (Merck Millipore, Burlington, MA). This was diluted with 15 mL binding buffer (8 M urea, 20 mM Na_2_H_2_PO_4_, 500 mM NaCl, 100 mM sucrose, 100 mM non-detergent sulfobetaines, 0.05% polyethylene glycol, 4 mM reduced glutathione, 0.04 mM oxidized glutathione and SIGMAFAST protease inhibitor cocktail at pH 7.8). The 1 mL HisTrap HP Ni-NTA column (GE Healthcare, Little Chalfont, UK) was connected to the AKTA Fast Protein Liquid Chromatography (FPLC) (GE Healthcare, Little Chalfont, UK) and equilibrated with 10 mL binding buffer at a flow rate of 1 mL/ min. The denatured protein was then loaded on to the column at a flow rate of 0.2 mL/ min. Once bound, the denatured protein was refolded by a gradual linear dilution of the 8 M urea to 0 M urea in refolding buffer (20 mM Na_2_H_2_PO_4_, 500 mM NaCl, 500 mM sucrose, 100 mM non-detergent sulfobetaines, 0.05% PEG, 4 mM reduced glutathione, 0.04 mM oxidized glutathione and SIGMAFAST protease inhibitor cocktail at pH 7.8) at a flow rate of 1 mL/ min for 50 column volumes. After renaturation, the column was washed with 10 column volumes of refolding buffer. Finally, the protein was eluted in a linear gradient for 20 column volumes with elution buffer (20 mM Na_2_H_2_PO_4_, 500 mM NaCl, 500 mM sucrose, 500 mM imidazole, 100 mM NDSB, 0.05% PEG, 4 mM reduced glutathione, 0.04 mM oxidized glutathione and SIGMAFAST protease inhibitor cocktail at pH 7.8). Fractions containing the recombinant protein were pooled then de-salted and concentrated using the Amicon Ultra 15 Centrifugal Filter Unit, 15 kDa NMWL according to the manufacturer’s instructions (Merck Millipore, Burlington, MA). The protein concentration was determined by the Bradford method (Bradford, 1976) and the recombinant protein was stored at -20°C.

Bradford, M. M. (1976) A rapid and sensitive method for the quantitation of microgram quantities of protein utilizing the principle of protein-dye binding, *Anal Biochem.* 72, 248-54.

**Supplementary Table 1: List of *Arabidopsis thaliana* candidate ACs:**

**Pattern Matching (PatMatch) search parameters**

Search pattern [R]X(5,20)[RKS][YFW][DE][VIL]X(4)[VIL]X(4)[KR]X(1,3)[DE]

Dataset searched TAIR10 Proteins (protein)

**Retrieved hits:**

**Gene: Description: Start End Hit pattern**

AT1G15290 TPR-like 534 557 RVSSVRPIRWELGSTWVQHLQKKE

AT1G22260 ZYP1A 558 586 RNDQAINEIRRKYDVEKHEIINSEKDKVE

AT1G22275 ZYP1B 558 586 RNDQAINEIRRKYDVEKHEIINSEKDKVE

AT1G26580 Not annotated 408 433 RLNVEDDSCTSFELARNAVNCAEKDE

AT2G25660 EMB2410 1273 1307 RLEVMITVEKTILEQSNSRYELQGEYVLPGSRDRD

AT3G11580 NGAL2, SOD7 110 144 RYVKDKHLDAGDVVFFQRHRFDLHRLFIGWRRRGE

AT3G17320 FOA1 296 321 REEKLAVLYQRWDITWTGIWISNKIE

AT3G18035 HON4 363 399 RKIGTSVTTGTQDSGELKKKFDIFQEKVKEIVKVLKD

AT3G20130 CYP705A22, GPS1 235 272 RQLQKLGISLFKKDIMGVSNKFDVLLEKVLVEHREKPE

AT3G57980 DNA-binding bromodomain 83 109 RVDELRREVERYDLSISSLQLKVKTLE

AT4G33530 KUP5 74 96 RTSPAVDSFDVDALEIPGTQKNE

AT5G09400 KUP7 73 95 RTGPRVDSFDVEALEVPGAPRND

AT5G36200 F-box & interact. domains 275 300 REEQLAVLIKRFDLWRMEIWVTTKIE

AT5G64370 BETA-UP, PYD3 364 392 RQYKDKWGFRMTARYEVYADLLAKYIKPD

**Supplementary Figure 1**

**A**

WT

Induced

Un-

induced

*cyaA* +

ATKUP5

*cyaA*

mutant

WT

Induced

Un-

induced

*cyaA* +

ATKUP5

S81P

*cyaA*

mutant

**B**


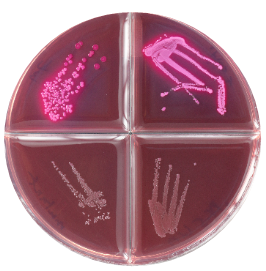


WT

Un-

induced

Induced

Un-

induced

Induced

*cyaA* +

AtKUP5^1-104^

*cyaA*

mutant


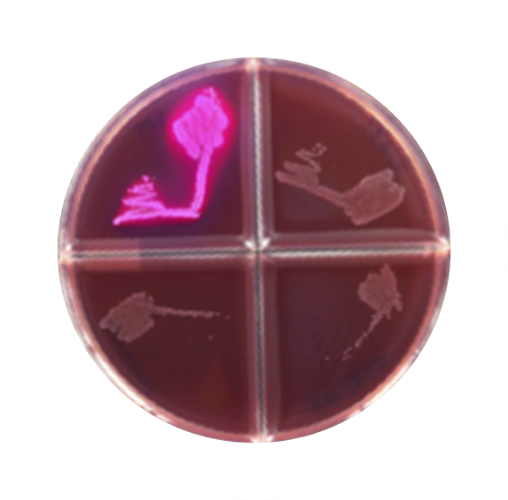


*cyaA* +

AtKUP5^1-104^

S81P/D83T

*cyaA*

mutant

WT

**Functional complementation of an AC deficient *E. coli* mutant with AtKUP5.** A, full length AtKUP5 complements the *E. coli* *cyaA* mutant (SP850) as indicated by the growth of red colonies on MacConkey agar (upper panel). The mutated full length AtKUP5 S81P, was unable to rescue the *cyaA* mutant (lower panel). B, the AtKUP5 AC domain (AtKUP5^1-104^) complements the *E. coli* *cyaA* mutant (SP850) as indicated by the growth of red colonies on MacConkey agar (upper panel). The mutated AC domain, AtKUP5^1-104^ S81P/D83T, was unable to rescue the *cyaA* mutant (lower panel).

**Supplementary Figure 2**

**A** **B**


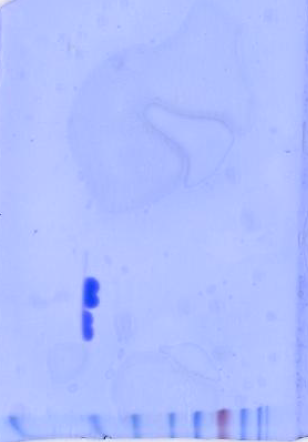


kD

72-

55-

43-

34-

26-

17-

L

1 2

2

1

L

Size (bp)

600-

500-

400-


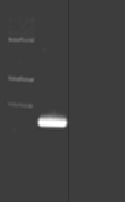


← 24 kDa

← 315 bp

**Cloning and expression of ATKUP5^1-104^.** A, Agarose gel electrophoresis analysis of the *AtKUP5^1-104^* clone. Lane L is the GeneRuler 100 bp (ThermoFisher Scientific, Waltham, MA) molecular weight marker. Lanes 1 and 2 are the amplified *ATKUP5^1-104^* PCR product and negative control, respectively. B, SDS-PAGE profile of the recombinant AtKUP5^1-104^ protein purified under denaturing conditions and refolded by FPLC. Lane L is the PageRuler pre-stained protein ladder 10-180 kDa (ThermoFisher Scientific, Waltham, MA). Lanes 1 and 2 indicate a band of the size expected for AtKUP5^1-104^.
